# ROS Mediate xCT-Dependent Cell Death in Human Breast Cancer Cells under Glucose Deprivation

**DOI:** 10.3390/cells9071598

**Published:** 2020-07-01

**Authors:** Mei-Chun Chen, Li-Lin Hsu, Sheng-Fan Wang, Chih-Yi Hsu, Hsin-Chen Lee, Ling-Ming Tseng

**Affiliations:** 1 Department and Institute of Pharmacology, School of Medicine, National Yang-Ming University, Taipei 112, Taiwan; mcchen23@vghtpe.gov.tw (M.-C.C.); lindahsu0807@yahoo.com.tw (L.-L.H.); sfwang5@vghtpe.gov.tw (S.-F.W.); 2 Division of Plastic and Reconstructive Surgery, Department of Surgery, Taipei Veterans General Hospital, Taipei 112, Taiwan; 3 Department of Surgery, School of Medicine, National Yang-Ming University, Taipei 112, Taiwan; 4 Department of Pharmacy, Taipei Veterans General Hospital, Taipei 112, Taiwan; 5 Department of Pathology and Laboratory Medicine, Taipei Veterans General Hospital, Taipei 112, Taiwan; cyhsu@vghtpe.gov.tw; 6 Taipei-Veterans General Hospital, Comprehensive Breast Health Center, Taipei 112, Taiwan; 7 Division of General Surgery, Department of Surgery, Taipei Veterans General Hospital, Taipei 112, Taiwan

**Keywords:** breast cancer, xCT, ROS, glucose dependency

## Abstract

xCT, also known as solute carrier family 7 member 11 (SLC7A11), the light chain of the cystine/glutamate antiporter, is positively correlated with cancer progression due to antioxidant function. During glucose deprivation, the overexpression of xCT does not protect cancer cells but instead promotes cell death. Further understanding the mechanism of glucose deprivation-induced cell death is important for developing anticancer treatments targeting the glucose metabolism. In this study, we found that breast cancer cells with a high expression of xCT demonstrated increased levels of reactive oxygen species (ROS) and were more sensitive to glucose deprivation than the cells with a low expression of xCT. However, AMP-activated protein kinase (AMPK) did not significantly affect glucose-deprivation-induced cell death. The antioxidant N-acetyl-cysteine prevented glucose-deprivation-induced cell death, and the glutathione biosynthesis inhibitor L-buthionine-S, R-sulfoximine enhanced glucose-deprivation-induced cell death. The inhibition of xCT by sulfasalazine or a knockdown of xCT reduced the glucose-deprivation-increased ROS levels and glucose-deprivation-induced cell death. Glucose deprivation reduced the intracellular glutamate, and supplementation with α-ketoglutarate prevented the glucose-deprivation-increased ROS levels and rescued cell death. The knockdown of sirtuin-3 (SIRT3) further enhanced the ROS levels, and promoted xCT-related cell death after glucose deprivation. In conclusion, our results suggested that ROS play a critical role in xCT-dependent cell death in breast cancer cells under glucose deprivation.

## 1. Introduction

Breast cancer is the most common malignancy and the leading cause of cancer death in women [1]. Although developments of antiestrogen (tamoxifen and aromatase inhibitors) and trastuzumab therapy (Herceptin) have benefited breast cancer patients, metastasis and recurrent cases still affect patient survival, in particular for patients with triple-negative breast cancer (TNBC) [2,3]. Therefore, more targeted, less toxic therapies for breast cancer are urgently needed.

Cancer cell proliferation requires excess nutrients compared with bioenergetics needs and shunts metabolites into pathways that support biosynthesis [4,5]. The deregulated cellular energetics and metabolic reprogramming were identified as cancer hallmarks [6]. The metabolic alterations were also demonstrated in certain subtypes of breast cancer, such as TNBC [7]. Understanding the metabolism of cancer cells is helpful for the development of anticancer therapies.

Glucose is an important nutrient to provide sufficient energy, biosynthesis material, and reduction–oxidation (redox) substitutes [8]. Cancer cells have been characterized as exhibiting high glycolysis but low mitochondrial oxidative phosphorylation (OXPHOS) activity even in the presence of oxygen, known as the Warburg effect [9,10]. Recently, the solute carrier family 7 member 11 (SLC7A11), also called xCT, was found to be involved in the glucose dependency [11,12,13]. The xCT is the light chain of the system x_c_^₋^ a cystine/glutamate antiporter at the plasma membrane, and is responsible for cystine uptake through an exchange with intracellular glutamate [14]. The imported cystine is converted to cysteine, the precursor for the synthesis of glutathione (GSH), which is involved in the redox balance in the cytosol and mitochondria. The xCT expression was found to be associated with maintaining the intracellular GSH level by importing cystine and associated with reducing oxidative stress [14]. The upregulation of xCT was correlated with a poor response to treatment in gastric cancer [15], esophageal cancer [16], hepatocellular cancer [17], and breast cancer [18]. The detailed mechanism by which xCT expression regulates the glucose dependency remains underevaluated.

AMP-activated protein kinase (AMPK) is another important regulator for metabolic and energy homeostasis. In glucose-limiting conditions, the activation of AMPK increased mitochondrial biogenesis, supported oxidative metabolism, and maintained the cellular ATP pool to promote cell survival [19]. In addition, sirtuin-3 (SIRT3) is a mitochondrial NAD+-dependent deacetylase and is involved in the cellular response to calorie restriction adaptation through the deacetylation of the mitochondrial proteins responsible for regulating mitochondrial reactive oxygen species (mtROS) homeostasis and the tricarboxylic acid (TCA) cycle [20,21]. In animal models, SIRT3 deacetylated and activated the antioxidant defense system under caloric restriction [22]. The functions of AMPK and SIRT3 in xCT-mediated glucose dependency are still unclear.

In this study, we evaluated the role of xCT in glucose-deprivation-induced cell death in breast cancer cells during glucose deprivation. We investigated the functions of AMPK and SIRT3 in xCT-mediated glucose dependency.

## 2. Materials and Methods

### 2.1. Cell Culture

The human breast cancer cell lines MCF-7, MDA-MB-231, and Hs-578t, as well as human embryonic kidney cells (HEK293T), were grown in Dulbecco’s Modified Eagle’s Medium (DMEM; Gibco, Grand Island, NY, USA) with 10% fetal bovine serum (FBS; Gibco, Grand Island, NY, USA), 1% penicillin/streptomycin (P/S; Biological Industries, Cromwell, CT, USA), 1% nonessential amino acid (NEAA; Biological Industries, Cromwell, CT, USA), and 1% L-glutamine. An additional 1% insulin (Sigma-Aldrich, St. Louis, MO, USA) was added in the cultured medium for Hs-578t. The MDA-MB-231 cells with a stable knockdown of SIRT3 were grown in DMEM with 1 μg/mL puromycin (Sigma-Aldrich, St. Louis, MO, USA). HCC-1937 was maintained in RPMI medium (Gibco, Grand Island, NY, USA) with 10% FBS and 1% P/S. The cells were cultured in a humidified incubator equilibrated at 37 °C in 5% CO_2_. For the glucose deprivation experiments, the cells were cultured in glucose-free DMEM (Gibco, Grand Island, NY, USA) or glucose-free RPMI medium (Gibco, Grand Island, NY, USA) with or without glucose (25 mM) (Sigma-Aldrich, St. Louis, MO, USA) for 24 h.

### 2.2. Propidium Iodide (PI) Exclusion Assay

The cells (2 × 10^5^ cells/well) were seeded in a six-well culture plate. The next day, the medium was changed to glucose-free DMEM or glucose-containing DMEM for different treatments. After 24 h, the cells were collected and resuspended in PBS with 5 mg/mL PI (Sigma-Aldrich, St. Louis, MO, USA). The PI fluorescence intensity at the FL1 was determined by flow cytometry (FACS Calibur flow cytometer, Becton Dickinson, Franklin Lakes, NJ, USA). A minimum of 20,000 cells were collected. The data were evaluated by Cell Quest software (Becton Dickinson, San Jose, CA, USA).

### 2.3. Intracellular ROS and Mitochondrial ROS Measurement

The cells (2 × 10^5^ cells/well) were seeded in a six-well culture plate. The next day, the medium was changed to glucose-free DMEM or glucose-containing DMEM for different treatments. After 24 h, the cells were incubated with 10 μM dichlorodihydro-fluorescein diacetate (DCFH-dA) for 30 min or 10 μM MitoSOX Red for 10 min. Then, the cells were collected and resuspended in PBS. The DCF fluorescence intensity at the FL1 and the MitoSOX Red fluorescence intensity at the FL2 were determined by flow cytometry. A minimum of 20,000 cells were collected. The data were evaluated by Cell Quest software. The DCF and MitoSOX Red were purchased from Molecular Probes^TM^, Invitrogen^TM^, and Thermo Fisher Scientific (Eugene, OR, USA).

### 2.4. Real-Time Polymerase Chain Reaction (PCR)

The cellular RNA was purified with TRIzol reagent (Invitrogen, Carlsbad, CA, USA). The method utilized acid-guanidinium thiocyanate and phenol/chloroform extraction. The RNA was reverse-transcribed into cDNA using a RevertAid^TM^ reverse transcriptase kit (Thermo Fisher Scientific, Waltham, MA, USA). Then, real-time PCR was performed with a StepOne^TM^ System (Applied Biosystems^TM^ real-time PCR Instrument, Thermo Fisher Scientific) and KAPA SYBR FAST qPCR Kit (Kapa Biosystems, Wilmington, MA, USA). The denatured condition was 95 °C for 3 min. Forty-five cycles, at 95 °C for 3 s and 60 °C for 30 s, were run. The relative gene expression levels were determined by the 2^−ΔΔCt^ method. The data was normalized to the expression of glyceraldehyde 3-phosphate dehydrogenase (GAPDH). The primer sequences were GAPDH, forward: 5′-CCGTCTAGAAAAACCTGCC-3′, reverse: 5′-GCCAAATTCGTTGTCATACC-3′; xCT, forward: 5′-TCATTGGAGCAGGAATCTTCA-3′, reverse: 5′-TTCAGCATAAGACAAAGCTCCA-3′.

### 2.5. Western Blot Analysis

Proteins (20 μg), from cell lysates, were separated by 8–12% SDS-polyacrylamide gel electrophoresis and transferred onto polyvinylidene difluoride membranes (Biotrace^TM^, PALL Life sciences, Ann Arbor, MI, USA). The sample membrane was immunoblotted with primary and secondary antibodies for at least 16 h. Signals from the antibody–protein conjugates were detected by the chemiluminescence kit (Immobilon Western Chemiluminescence HRP Substrates, Merck-Millipore, Billerica, MA, USA). The images of the relative bands were analyzed by the luminescence/fluorescence imaging system (GE healthcare) and MultiGauge image analysis software version 3.0 (Fujifilm, Stockholm, Sweden). The α-tubulin antibody was purchased from Invitrogen^TM^, Thermo Fisher Scientific (Carlsbad, CA, USA). Antibodies against xCT, SIRT3, AMPK, p-AMPK, acetyl-CoA carboxylase (ACC), p-ACC, and acetylated-lysine Ac-K-103 were purchased from Cell Signaling Technology (Beverly, MA, USA).

### 2.6. Small Interfering RNA (siRNA)-Mediated Genetic Knockdown

The cells (2 × 10^5^ cells) were seeded in a six-well culture plate. After resting overnight, lipofectamine RNAi MAX reagent (Invitrogen^TM^, Thermo Fisher Scientific, Carlsbad, CA, USA) and the indicated concentration of siRNA were added in serum-free DMEM. After 5 min at room temperature, the siRNA-lipid complex was formed. The complex was added to antibiotic-free medium. After 48 h, the cells were collected for further experiments. We performed Western blotting to confirm the effect of siRNA-mediated genetic knockdowns. Nontarget (scramble) and SLC7A11(xCT) siRNAs were purchased from GE healthcare Dharmacon (Lafayette, CO, USA).

### 2.7. xCT Overexpression

HEK293T cells (2 × 10^5^ cells/well) were seeded in a six-well plate. The next day, 2 μg/well pCMV6-xCT or empty vector (EV) pCMV6 was mixed with 2 μL turboFect Transfection reagent (Thermo Fisher Scientific, Waltham, MA, USA) in antibiotic/serum-free DMEM for 15 min. The mixture was used to transfect cells cultured in antibiotic-free DMEM. After 24 h, the cells could be used for further experiments. The transfect effect was evaluated using a Western blot.

### 2.8. Intracellular Glutamate Measurement

The cells were seeded in a 10 cm dish at a density of 6 × 10^5^ cells overnight. The medium was transferred with the glucose-free medium or the glucose-containing medium for 1 h. The intracellular level of glutamate was determined using a Glutamate Colorimetric Assay Kit (BioVision, Milpitas, CA, USA) according to the manufacturer’s protocol. Briefly, cells (1 × 10^6^ cells) were homogenized in 100 μL assay buffer. After centrifugation at 13,000× *g* for 10 min, 100 μL assay buffer was added to the sample. Then, 20 μL of assay buffer was added, and the sample was incubated for 30 min at 37 °C. The intracellular glutamate was measured using a microplate reader (Tecan Austria GmbH, Grödig, Austria) for the OD at 450 nm.

### 2.9. Statistical Analysis

The data are presented as the mean ± the SEM of the results from three independent experiments in triplicate. GraphPad PRISM software version 6 (GraphPad Software, La Jolla, CA, USA) was used for the statistical analysis. The statistical significance of the differences between two groups was analyzed by an unpaired Student’s t-test. The significance level was set at less than 0.05.

## 3. Results

### 3.1. Glucose Deprivation Increased Intracellular ROS Levels and Induced Cell Death in Human Breast Cancer Cells

**Figure 1:**
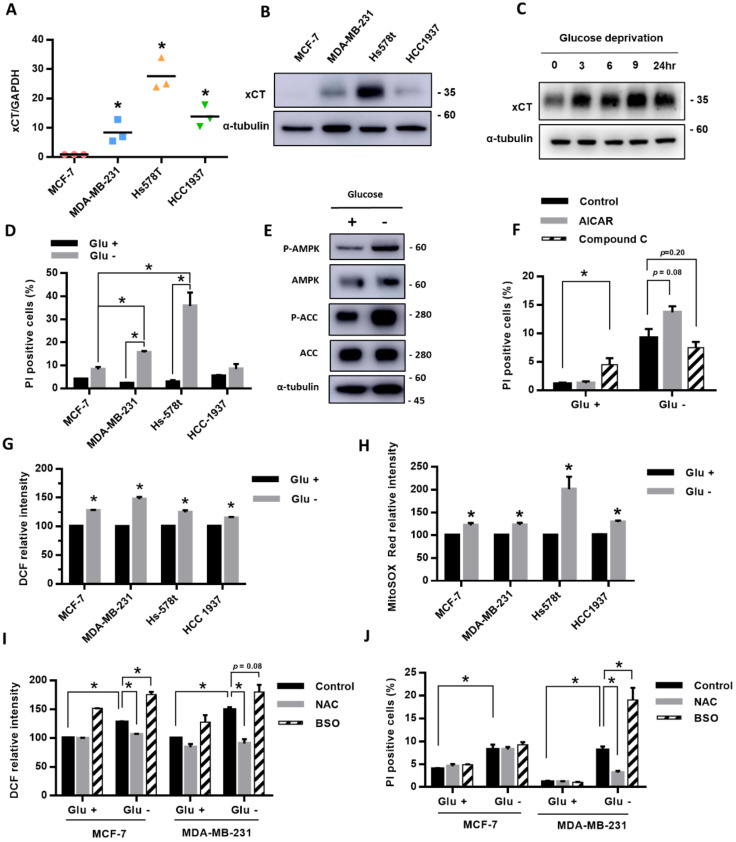
The breast cancer cells with high expression of xCT had higher ROS levels and a higher cell death rate under glucose deprivation. (**A**,**B**) The expression of xCT in breast cancer cell lines (MCF-7, MDA-MB-231, Hs-578t, and HCC-1937) in the normal culture medium was detected by real-time RT-PCR (**A**) and Western blot (**B**). The relative expression level of xCT of MCF-7 was set as 1. (**C**) The protein levels of xCT after glucose deprivation for 3, 6, 9, and 24 h in MDA-MB-231 cells were detected using a Western blot. (**D**)The four breast cancer cell lines were cultured in the medium with or without glucose (25 mM) for 24 h. The cell death rate was evaluated by the flow cytometry with PI exclusion assay. (**E**,**F**) MDA-MB-231 cells were treated with or without glucose deprivation for 24 h. The protein levels of the AMPK pathway were detected using a Western blot (**E**). The cells were cotreated with the AMPK activator 5-aminoimidazole-4-carboxamide ribonucleotide (AICAR, 1 mM) or the AMPK inhibitor Compound C (Com C, 10 μM). The cell death rate was detected using flow cytometry with a PI exclusion assay (**F**). (**G**,**H**) The levels of intracellular ROS and mtROS were detected using flow cytometry with DCFH-dA staining (**G**) and mitoSOX Red dye (**H**). The measured value of ROS from the cells cultured in the glucose-containing medium (control) was normalized as 100%. (**I**) The MCF-7 and MDA-MB-231 cells were cultured in the medium with or without glucose and cotreated with the antioxidant N-acetyl-cysteine (NAC, 1 mM) or the glutathione biosynthesis inhibitor L-buthionine-S, R-sulfoximine (BSO, 150 μM). The levels of intracellular ROS after NAC or BSO treatment were detected using flow cytometry with DCFH-dA staining. (**J**) The cell death rate was evaluated by the flow cytometry with a PI exclusion assay. The data are presented as the mean ± SEM of the results from three independent experiments in triplicate. *n* = 3. * (**A**,**D**,**F**–**J**): *p* < 0.05. Glu +: medium containing 25 mM glucose. Glu -: Glucose-free medium.

We first compared the expression of xCT in four breast cancer cell lines, MCF-7, MDA-MB-231, Hs-578t, and HCC-1937, and we found that MDA-MB-231, Hs-578t, and HCC1937 cells had higher gene and protein expressions of xCT than MCF-7 cells (Figure 1A,B). After treatment with glucose deprivation, the protein level of xCT was gradually elevated with time (Figure 1C). During glucose deprivation, the cell death rate in MDA-MB-231 and Hs-578t cell lines was higher than in MCF-7 cells and also higher than in cells without glucose deprivation (Figure 1D). The results suggest that the high-xCT-expressed cells were more sensitive to glucose deprivation than the low-xCT-expressed cells.

To evaluate whether the energy sensor AMPK is involved in the glucose-deprivation-induced cell death of the breast cancer cells, we examined the effect of glucose deprivation on the AMPK activation. We found that the phosphorylation levels of AMPK and its downstream target acetyl-CoA carboxylase (ACC) were significantly increased in MDA-MB-231 cells under glucose deprivation (Figure 1E). The results suggested that glucose deprivation was able to activate AMPK in the breast cancer cells. The AMPK activator 5-aminoimidazole-4-carboxamide ribonucleotide (AICAR) enhanced the glucose-deprivation-induced cell death, while the AMPK inhibitor Compound C attenuated the glucose-deprivation-induced cell death (Figure 1F). However, these effects did not reach statistical significance.

To explore the role of ROS in the glucose-deprivation-induced cell death in the high-xCT-expressed cells, we evaluated the levels of intracellular ROS and mtROS of the breast cancer cells after glucose deprivation. The results revealed that glucose deprivation significantly increased both the intracellular ROS and mtROS levels of the breast cancer cells (Figure 1G,H). We used MDA-MB-231 as the representative cell line with a higher expression of xCT due to a suitable cell death rate under glucose deprivation, and this could be treated with another stress condition without a high amount of cell death. Treatment with the antioxidant N-acetylcysteine (NAC) prevented the glucose-deprivation-increased ROS levels and glucose-deprivation-induced cell death (Figure 1I,J). In addition, treatment with the GSH biosynthesis inhibitor L-buthionine-S, R-sulfoximine (BSO) significantly enhanced the glucose-deprivation-increased ROS levels and glucose-deprivation-induced cell death (Figure 1I,J). MDA-MB-231 cells were more sensitive to glucose deprivation than the MCF-7 cells. The results suggested that ROS may be involved in glucose-deprivation-induced cell death.

### 3.2. Inhibition or Knockdown of xCT Prevented the Glucose-Deprivation-Increased ROS Levels and Rescued the Glucose-Deprivation-Induced Cell Death

**Figure 2:**
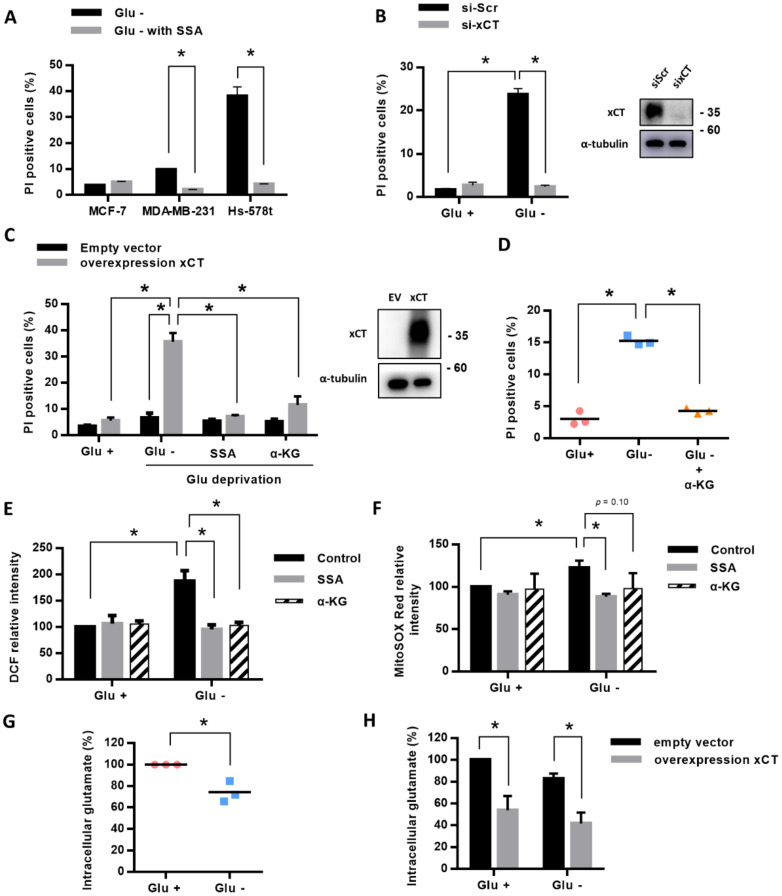
Inhibition of xCT or supplementation with α-KG reduced the ROS levels and rescued cell death in the cells under glucose deprivation. (**A**) Breast cancer cell lines were cultured in the glucose-free medium with or without sulfasalazine (SSA, xCT inhibitor, 500 μM) for 24 h. The cell death rate was detected using flow cytometry with a PI exclusion assay. (**B**) The MDA-MB-231 cells were treated with si-RNAs for scramble (si-Scr) or xCT knockdown (si-xCT) for 48 h. (**C**) HEK293t cells were transfected with xCT-pCMV6 (pxCT) overexpression plasmid or empty vector (EV) pCMV6 for 48 h. Then, the HEK293t cells with plasmid transfection were treated with or without glucose deprivation for 24 h. SSA (500 μM) or dimethyl α-ketoglutarate (α-KG) (4 mM) was cotreated in the groups of glucose deprivation in the HEK293t cells. The cell death rate was detected using flow cytometry with PI exclusion assay. (**D**) The MDA-MB-231 cells were cultured in glucose-containing medium or in glucose-free medium with α-KG for 24 h. The cell death rate was detected using flow cytometry with a PI exclusion assay. (**E**,**F**) The levels of intracellular ROS and mtROS were detected using flow cytometry with DCFH-dA staining (**E**) and mitoSOX Red dye (**F**). The measured value of ROS from the cells cultured in the glucose-containing medium (control) was normalized as 100%. (**G**,**H**) A glutamate assay kit was used to detect the intracellular glutamate level after treating with or without glucose deprivation for 1 h in MDA-MB-231 cells cultured in glutamine-free medium (**G**) or in the HEK293t cells with xCT-pCMV6 (pxCT) overexpression plasmid or empty vector (EV) pCMV6 transfection (**H**). The data are presented as the mean ± SEM of the results from three independent experiments in triplicate. n = 3. * (**A**–**H**): *p* < 0.05. Glu +: medium containing 25 mM glucose. Glu -: glucose-free medium.

To evaluate whether the xCT expression is essential for the glucose-deprivation-increased ROS levels and glucose-deprivation-induced cell death, we treated the MDA-MB-231 and Hs578t cells with the xCT inhibitor sulfasalazine (SSA) and found that inhibition of the xCT function significantly rescued the glucose-deprivation-induced cell death (Figure 2A). We used si-RNAs against xCT to knockdown the xCT expression and found that the knockdown of xCT expression significantly inhibited glucose-deprivation-induced cell death (Figure 2B).

In addition, we found that the overexpression of xCT in HEK293t cells significantly induced cell death after glucose deprivation, and SSA was able to prevent glucose-deprivation-induced cell death (Figure 2C). We further evaluated whether the xCT expression is important for glucose-deprivation-increased ROS levels, and found that SSA was able to significantly reduce the intracellular ROS (Figure 2E) and mtROS (Figure 2F) levels that were induced by glucose deprivation. These results suggested that the xCT expression is essential for the glucose-deprivation-increased ROS levels and glucose-deprivation-induced cell death.

### 3.3. xCT Accelerated the Glucose-Deprivation-Decreased Intracellular Glutamate and Supplementation with α-KG Prevented the Glucose-Deprivation-Increased ROS Levels and Induced Cell Death

As the function of xCT is responsible for cystine uptake through exchange with intracellular glutamate, our previous results suggested that the xCT-mediated cystine uptake for GSH biosynthesis might not be sufficient to prevent glucose-deprivation-induced cell death. We thus proposed that the increased export of intracellular glutamate might contribute to glucose-deprivation-induced cell death.

To examine this hypothesis, we detected the effect of glucose deprivation on intracellular glutamate levels and found that glucose deprivation significantly reduced the intracellular glutamate levels in MDA-MB-231 cells and HEK293t cells (Figure 2G,H). The overexpression of xCT in HEK293t cells significantly reduced the intracellular glutamate levels in both the glucose-containing medium and the glucose-free medium (Figure 2H). These results suggested that xCT might accelerate the glucose-deprivation-reduced glutamate levels. 

Intracellular glutamate may be transported into mitochondria and be converted to α-ketoglutarate (α-KG), which enters the tricarboxylic acid (TCA) cycle. To determine whether the decrease in intracellular glutamate level might contribute to glucose-deprivation-induced cell death through reducing the carbon source of the TCA cycle, we treated with permeable α-KG, dimethyl-α-KG, and found that supplementation with the α-KG was able to rescue the glucose-deprivation-induced cell death in the HEK293t cells with xCT overexpression (Figure 2C) and MDA-MB-231 cells (Figure 2D). The supplementation with the α-KG significantly prevented the intracellular ROS (Figure 2E) and mtROS (Figure 2F) levels that were induced by glucose deprivation. These results suggested that the xCT mediates the glucose-deprivation-increased ROS levels and glucose-deprivation-induced cell death through reducing the intracellular glutamate levels.

### 3.4. Knockdown of SIRT3 Enhanced the Glucose-Deprivation-Increased ROS Levels and Glucose-Deprivation-Induced Cell Death

**Figure 3:**
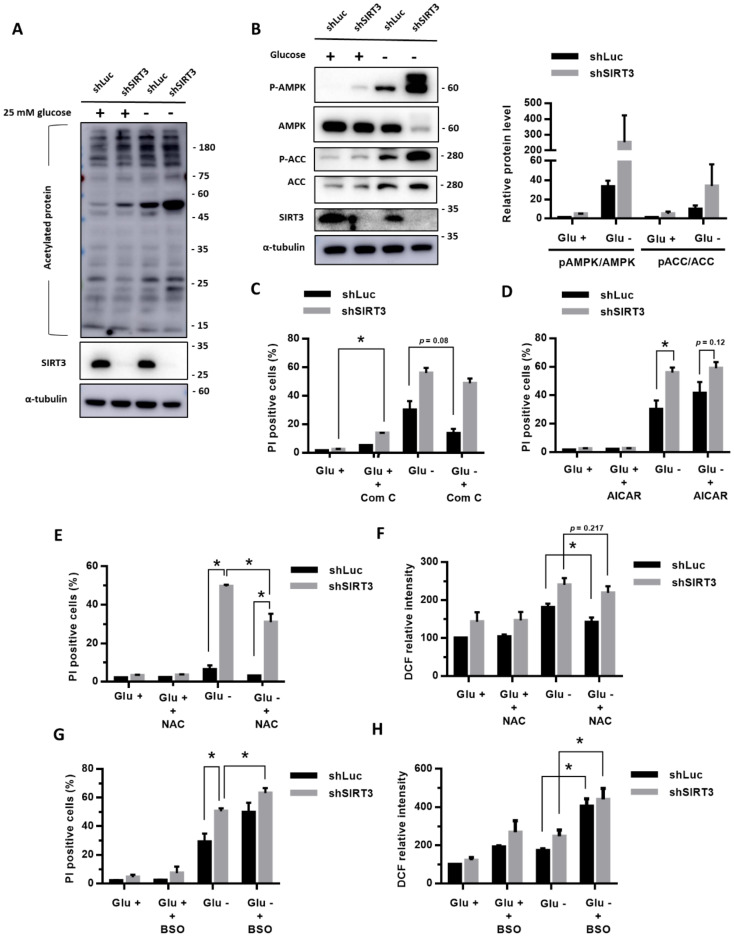
Knockdown of SIRT3 enhanced the ROS levels and promoted cell death in the cells under glucose deprivation. (**A**–**D**) The MDA-MB-231 cells with shSIRT3 to knockdown SIRT3 or control shLuc were treated with or without glucose deprivation for 24 h. (**A**) The level of acetylated protein was detected using a Western blot. (**B**) The protein levels of AMPK pathway were detected using a Western blot. (**C**,**D**) The cell death rate after cotreatment with Com C (10 μM) (**C**) or AICAR (1 mM) (**D**) for 24 h was detected using flow cytometry with a PI exclusion assay. (**E**–**H**) The antioxidant N-acetyl-cysteine (NAC) or the glutathione biosynthesis inhibitor L-buthionine-S, R-sulfoximine (BSO) was cotreated for 24 h. The cell death rate was evaluated using flow cytometry with a PI exclusion assay (**E**,**G**). The intracellular ROS levels were detected using flow cytometry with DCFH-dA staining (**F**,**H**). The measured value of the DCF intensity from the cells with shLuc in glucose-containing medium was normalized as 100%. The data are presented as the mean ± SEM of the results from three independent experiments in triplicate. *n* = 3. * (**C**–**H**): *p* < 0.05. Glu +: medium containing 25 mM glucose. Glu -: glucose-free medium.

Based on the importance of SIRT3 in the cellular response to nutrition deficiency, we evaluated whether SIRT3 is involved in the stress response of glucose deprivation. We used shRNA against SIRT3 to knockdown SIRT3 expression and selected the MDA-MB-231 cells with a stable expression of shSIRT3. Figure 3A shows that glucose deprivation increased the protein acetylation levels (Figure 3A). The levels of acetylated proteins in the MDA-MB-231 cells with a stable knockdown of SIRT3 (shSIRT3) were higher than the levels in the control cells with shLuc, confirming the deacetylation function of SIRT3 (Figure 3A).

In addition, we determined that the knockdown of SIRT3 enhanced the glucose-deprivation-increased phosphorylation levels of AMPK and ACC (Figure 3B), suggesting that SIRT3 knockdown enhances the glucose-deprivation-induced AMPK activation. In the SIRT3 knockdown cells, treatments with Compound C partly reduced the glucose-deprivation-induced cell death (Figure 3C), while treatments with AICAR enhanced the glucose-deprivation-induced cell death (Figure 3D). However, these effects did not reach statistical significance. These results suggested that AMPK activation might not be the major regulator in glucose-deprivation-induced cell death.

Importantly, we found that knockdown of SIRT3 significantly increased the cell death rate (Figure 3E) and the intracellular ROS levels (Figure 3F) under glucose deprivation. In addition, NAC treatments significantly reduced the glucose-deprivation-induced cell death in both the SIRT3-knockdown (shSIRT3) cells and the control (shLuc) cells (Figure 3E), though the NAC treatments mildly decreased the intracellular ROS levels (*p* = 0.217, Figure 3F). On the other hand, BSO treatments significantly enhanced the glucose-deprivation-induced cell death (Figure 3G) and glucose-deprivation-increased ROS levels in the cells with a stable shSIRT3 knockdown (shSIRT3) compared with the control (shLuc) cells (Figure 3H). These results suggested that SIRT3 may protect the cells from glucose-deprivation-increased ROS levels and glucose-deprivation-induced cell death.

### 3.5. Inhibition of xCT or Supplementation with α-KG Prevented the Effects of SIRT3 Knockdown on the Glucose-Deprivation-Increased ROS Levels and Glucose-Deprivation-Induced Cell Death

**Figure 4:**
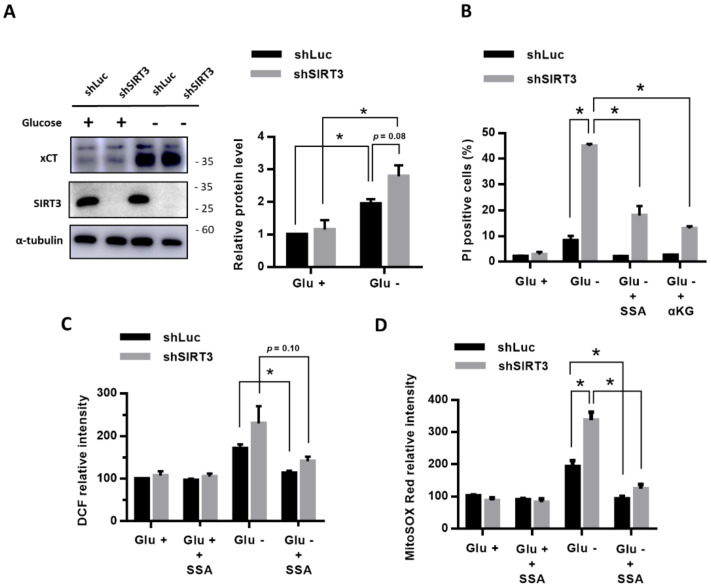
The inhibition of xCT or supplementation with α-KG prevented the effects of the SIRT3 knockdown on the glucose-deprivation-increased ROS levels and glucose-deprivation-induced cell death. (**A**) The MDA-MB-231 cells with shSIRT3 to knockdown SIRT3 or control shLuc were treated with or without glucose deprivation for 24 h. The protein levels of xCT and SIRT3 were detected using a Western blot. (**B**) The MDA-MB-231 cells with shLuc or shSIRT3 were treated with SSA or α-KG in glucose-free medium for 24 h. The cell death rate was evaluated using flow cytometry with a PI exclusion assay. (**C**,**D**) The MDA-MB-231 cells with shLuc or shSIRT3 were treated with or without glucose deprivation and SSA for 24 h. The levels of intracellular ROS and mtROS were detected using flow cytometry with DCFH-dA staining (**C**) and mitoSOX Red dye (**D**), respectively. The measured value from the cells with shLuc in the glucose-containing medium was normalized as 100%. The data are presented as the mean ± SEM of the results from three independent experiments in triplicate. *n* = 3. * (**A**–**D**): *p* < 0.05. Glu +: medium containing 25 mM glucose. Glu -: glucose-free medium.

We further evaluated whether the knockdown of SIRT3 affects the xCT expression under glucose deprivation, and found that, under glucose deprivation, the MDA-MB-231 cells with a knockdown of SIRT3 (shSIRT3) did not have significantly higher xCT protein levels than the control cells with shLuc (*p* = 0.08) (Figure 4A). The results revealed that a knockdown of SIRT3 did not enhance the glucose-deprivation-increased xCT expression.

To examine whether the function of xCT is also essential for the glucose-deprivation-increased ROS levels and glucose-deprivation-induced cell death of the breast cancer cells with an SIRT3 knockdown, we treated the SIRT3 knockdown cells with the xCT inhibitor SSA. We found that the SSA treatments were able to significantly prevent the glucose-deprivation-induced cell death (Figure 4B). The SSA treatments significantly reduced the glucose-deprivation-increased intracellular ROS levels (Figure 4C) and mitochondrial ROS levels (Figure 4D) of the SIRT3 knockdown cells as well as the control (shLuc) cells. In addition, supplementation with α-KG was able to significantly reduce the glucose-deprivation-induced cell death of the SIRT3 knockdown cells (Figure 4B). These results revealed that the function of xCT is involved in the glucose-deprivation-increased ROS levels and glucose-deprivation-induced cell death of the SIRT3 knockdown cells.

These results show that SIRT3 has protective effects during glucose starvation. The knockdown of SIRT3 under glucose deprivation promoted the xCT-related glucose dependency through the upregulation of xCT and increased ROS levels, particularly mtROS.

## 4. Discussion

Our present results clearly demonstrated that the breast cancer cells with a high expression of xCT were more susceptible to glucose deprivation than the cells with low expression of xCT. The increased ROS levels contributed to the xCT-dependent cell death of human breast cancer cells under glucose deprivation. In addition, SIRT3 might play a protective role in the glucose-deprivation-increased ROS levels, in particular mtROS, and in the glucose-deprivation-induced cell death of breast cancer cells with a high expression of xCT.

**Figure 5:**
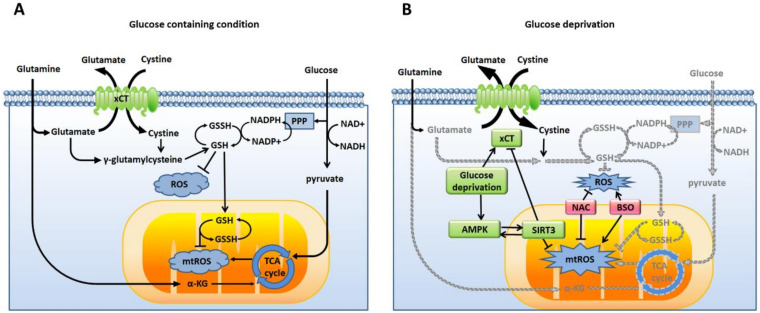
ROS mediate the xCT-related glucose dependency. (**A**) When nutrients are sufficient, cancer cells use glucose and glutamine to produce energy and antioxidants to balance the intracellular ROS and mtROS. (**B**) During glucose deprivation, the production of energy and antioxidants is decreased (gray). xCT upregulation promotes the import of cystine by exchanging glutamate. AMPK is also activated to regulate the antioxidant function of SIRT3. The knockdown of SIRT3 increases the expression of xCT, increases the ROS levels, and enhances the glucose dependency of cancer cells. The antioxidant N-acetyl-cysteine (NAC) is able to prevent glucose-deprivation-induced cell death. The glutathione biosynthesis inhibitor L-buthionine-S, R-sulfoximine (BSO) enhances glucose-deprivation-induced cell death. These results revealed that ROS are involved in xCT-related glucose dependency.

Glucose and glutamine are two major nutrients for cancer cells [23]. Glucose is the major carbon source to form ATP by glycolysis (the Warburg effect) or coupled with oxidative phosphorylation via the TCA cycle in the mitochondria. Glucose is also involved in the formation of NADPH, an antioxidant, through the pentose phosphate pathway (PPP). On the other hand, after uptake by cells, glutamine is converted to glutamate and, then, is further converted to α-KG, a key intermediate in the TCA cycle. Therefore, intracellular glutamine and glutamate are essential carbon sources to maintain the TCA cycle and oxidative phosphorylation in the mitochondria when glucose is limited. The system x_c_^₋^ is a cystine/glutamate antiporter at the plasma membrane, and is responsible for cysteine uptake by exchange with intracellular glutamate. xCT (SLC7A11) is the light chain of the system x_c_^₋^ and mediates the transport activity. The uptake of extracellular cystine, through exchanging intracellular glutamate, is the major source of cysteine to biosynthesize and maintain the intracellular GSH level, which is involved in the redox balance with NADPH. Therefore, glucose and glutamine both contribute to energy production and the redox balance (Figure 5A).

In our previous studies, cystine-deprivation-induced intracellular GSH degradation enhanced cell death in triple-negative breast cancer cells with high xCT expression [24]. The high expression of xCT and GSH biosynthesis were found to decrease the cisplatin-induced oxidative damage and contribute to cisplatin resistance in human cancer cells [15]. These findings supported the importance of xCT in redox regulation. The upregulation of xCT contributes to the use of glucose as the major carbon source but impairs the glutamate metabolism due to the decreased intracellular glutamate level. xCT was thus proposed to promote glucose addiction in cancer cells and to restrict nutrient flexibility [13]. Our present findings further supported that xCT is also a metabolic regulator.

Under glucose deprivation, the carbon source to form ATP and the redox balance might be shifted to highly depend on glutamine. Our results showed that the xCT expression (Figure 1C) and the energy sensor AMPK (Figure 1E) were both upregulated during glucose deprivation. However, the AMPK activation by glucose deprivation did not appear to have a significant effect on the cell survival (Figure 1F). On the other hand, glucose deprivation induced a decrease in the intracellular glutamate levels, increases in the intracellular and mitochondrial ROS levels, and cell death, which were highly dependent on the xCT function (Figure 2). The increased ROS levels were demonstrated to contribute to glucose-deprivation-induced cell death.

Supplementation with α-KG was found to control the ROS levels and rescue glucose-deprivation-induced cell death. Therefore, these results suggested that, under glucose deprivation, the high expression of xCT might cause breast cancer cells to highly consume intracellular glutamate for the exchange of cysteine to rescue the redox imbalance due to loss of the substrates of the PPP from glycolysis. In addition, the biosynthesis process from cystine to GSH could be inhibited by insufficient NADPH levels when the PPP is limited due to glucose deprivation. The high expression of xCT in cells could lead to a redox system collapse [25]. These findings suggest that xCT regulates glucose-deprivation-induced cell death through the redox balance and nutrient regulation.

SIRT3 is important in the regulation of mtROS and energy production. The transcription of *SIRT3* can be activated by oxidative stress or under nutrient deprivation [26]. SIRT3 activates manganese superoxide dismutase (MnSOD) by deacetylating MnSOD-K68 to reduce the ROS levels [26]. SIRT3 was also demonstrated to deacetylate isocitrate dehydrogenase 2 and enzymes in the TCA cycle and in the electron transport chain to regulate ATP production, fatty acid oxidation, glucose oxidation, and mitochondrial biogenesis [27,28,29]. The loss of SIRT3 may lead to increased ROS levels, genomic instability, and intracellular metabolism with increased glycolysis and decreased oxidative phosphorylation [30].

Our present results demonstrated that the knockdown of SIRT3 further increased the intracellular ROS and mtROS levels of breast cancer cells under glucose deprivation (Figure 4). The inhibition of xCT or supplementation with α-KG prevented the effects of the SIRT3 knockdown on the glucose-deprivation-increased ROS levels and glucose-deprivation-induced cell death (Figure 4). Therefore, these lines of evidence suggested that SIRT3 might play a protective role in the xCT-dependent cellular adaptation response to glucose deprivation (Figure 5B). Our results support that SIRT3 functions with a prosurvival role through the activation of the antioxidant defense system in human breast cancer cells during glucose deprivation.

AMPK is an energy sensor and is typically activated during metabolic stress conditions or under a high AMP/ATP ratio. When under caloric restriction, AMPK positively upregulated the expression of SIRT3 via activating the peroxisome proliferator-activated receptor-gamma coactivator (PGC-1α) or increasing the PGC-1α expression via the cyclic AMP response element binding protein [31,32]. SIRT3 also positively regulated the expression of AMPK via deacetylation of the liver kinase B-1 [33]. These form a positive feedback loop of SIRT3 and AMPK. SIRT3, then, activated the downstream target for redox balance and caloric restriction-related adaption responses. The increased mtROS could indirectly activate the AMPK pathway [34].

Our results revealed that the knockdown of SIRT3 enhanced the phosphorylation level of AMPK under glucose deprivation. The increased mtROS due to the knockdown of SIRT3 could contribute to the elevated phosphorylation level of AMPK. Under glucose deprivation, our data showed that the inhibitor or activator of AMPK could not change the cell death rate of the breast cancer cells with SIRT3 knockdown. The downstream antioxidant defense system of SIRT3 could not be activated by AMPK when SIRT3 was in knockdown. These results further supported that SIRT3 plays an important role in protecting cells from glucose-deprivation-induced cell death.

In conclusion, our results demonstrated that increased ROS levels contribute to the xCT-dependent cell death of human breast cancer cells under glucose deprivation. In addition, SIRT3 may play a protective role in the glucose-deprivation-increased ROS levels and glucose-deprivation-induced cell death of breast cancer cells with a high expression of xCT. These findings will improve our understanding of the role of xCT in breast cancer progression and will benefit the development of therapeutic strategies for human breast cancer therapy.

## References

[1] Bray F., Ferlay J., Soerjomataram I., Siegel R.L., Torre L.A., Jemal A. (2018). Global cancer statistics 2018: GLOBOCAN estimates of incidence and mortality worldwide for 36 cancers in 185 countries. CA Cancer J. Clin..

[2] Chacón R.D., Costanzo M.V. (2010). Triple-negative breast cancer. Breast Cancer Res..

[3] Dent R., Trudeau M., Pritchard K.I., Hanna W.M., Kahn H.K., Sawka C.A., Lickley L.A., Rawlinson E., Sun P., Narod S.A. (2007). Triple-negative breast cancer: Clinical features and patterns of recurrence. Clin. Cancer Res..

[4] DeBerardinis R.J., Lum J.J., Hatzivassiliou G., Thompson C.B. (2008). The biology of cancer: Metabolic reprogramming fuels cell growth and proliferation. Cell Metab..

[5] Cairns R.A., Harris I.S., Mak T.W. (2011). Regulation of cancer cell metabolism. Nat. Rev. Cancer.

[6] Hanahan D., Weinberg R.A. (2011). Hallmarks of cancer: The next generation. Cell.

[7] McCleland M.L., Adler A.S., Shang Y., Hunsaker T., Truong T., Peterson D., Torres E., Li L., Haley B., Stephan J.P. (2012). An integrated genomic screen identifies LDHB as an essential gene for triple-negative breast cancer. Cancer Res..

[8] Barbosa A.M., Martel F. (2020). Targeting glucose transporters for breast cancer therapy: The effect of natural and synthetic compounds. Cancers.

[9] Warburg O. (1956). On respiratory impairment in cancer cells. Science.

[10] Upadhyay M., Samal J., Kandpal M., Singh O.V., Vivekanandan P. (2013). The Warburg effect: Insights from the past decade. Pharmacol. Ther..

[11] Goji T., Takahara K., Negishi M., Katoh H. (2017). Cystine uptake through the cystine/glutamate antiporter xCT triggers glioblastoma cell death under glucose deprivation. J. Biol. Chem..

[12] Koppula P., Zhang Y., Shi J., Li W., Gan B. (2017). The glutamate/cystine antiporter SLC7A11/xCT enhances cancer cell dependency on glucose by exporting glutamate. J. Biol. Chem..

[13] Shin C.S., Mishra P., Watrous J.D., Carelli V., D’Aurelio M., Jain M., Chan D.C. (2017). The glutamate/cystine xCT antiporter antagonizes glutamine metabolism and reduces nutrient flexibility. Nat. Commun..

[14] Lo M., Wang Y.Z., Gout P.W. (2008). The x(c)- cystine/glutamate antiporter: A potential target for therapy of cancer and other diseases. J. Cell Physiol..

[15] Wang S.F., Wung C.H., Chen M.S., Chen C.F., Yin P.H., Yeh T.S., Chang Y.L., Chou Y.C., Hung H.H., Lee H.C. (2018). Activated integrated stress response induced by salubrinal promotes cisplatin resistance in human gastric cancer cells via enhanced xCT expression and glutathione biosynthesis. Int. J. Mol. Sci..

[16] Shiozaki A., Iitake D., Ichikawa D., Nakashima S., Fujiwara H., Okamoto K., Kubota T., Komatsu S., Kosuga T. (2014). xCT, component of cysteine/glutamate transporter, as an independent prognostic factor in human esophageal squamous cell carcinoma. J. Gastroenterol..

[17] Kinoshita H., Okabe H., Beppu T., Chikamoto A., Hayashi H., Imai K., Mima K., Nakagawa S., Ishimoto T., Miyake K. (2013). Cystine/glutamic acid transporter is a novel marker for predicting poor survival in patients with hepatocellulcar carcinoma. Oncol. Rep..

[18] Yang Y., Yee D. (2014). IGF-1 regulates redox status in breast cancer by activating the amino acid transport molecule xC-. Cancer Res..

[19] Chaube B., Malvi P., Singh S.V., Mohammad N., Viollet B., Bhat M.K. (2013). AMPK maintains energy homeostasis and survival in cancer cells via regulating p38/PGC-1α-mediated mitochondrial biogenesis. Cell Death. Discov..

[20] Bell E.L., Guarente L. (2011). The SirT3 divining rod points to oxidative stress. Mol. Cell.

[21] Herbert A.S., Dittenhafer-Reed K.E., Yu W., Bailey D.J., Selen E.S., Boersma M.D., Carson J.J., Tonelli M., Balloon A.J., Higbee A.J. (2013). Calorie restriction and SIRT3 trigger global reprogramming of the mitochondrial protein acetylome. Mol. Cell.

[22] Qiu X., Brown K., Hirschey M.D., Verdin E., Chen D. (2010). Calorie restriction reduces oxidative stress by SIRT3-mediated SOD2 activation. Cell Metab..

[23] Ward P.S., Thompson C.B. (2012). Metabolic reprogramming: A cancer hallmark even Warburg did not anticipate. Cancer Cell.

[24] Chen M.S., Wang S.F., Hsu C.Y., Yin P.H., Yeh T.S., Lee H.C., Tseng L.M. (2017). CHAC1 degradation of glutathione enhances cystine-starvation-induced necroptosis and ferroptosis in human triple negative breast cancer cells via the GCN2-eIF2α-ATF4 pathway. Oncotarget.

[25] Liu X., Olszewski K., Zhang Y., Lim E.W., Shi J., Zhang X., Zhang J., Lee H., Kopplula P., Lei G. (2020). Cystine transporter regulation of pentose phosphate pathway dependency and disulfide stress exposes a targetable metabolic vulnerability in cancer. Nat. Cell Biol..

[26] Chen Y., Zhang J., Lin Y., Lei Q., Guan K.L., Zhao S., Xiong Y. (2011). Tumour suppressor SIRT3 deacetylates and activates manganese superoxide dismutase to scavenge ROS. EMBO Rep..

[27] Someya S., Yu W., Hallows W.C., Xu J., Vann J.M., Leeuwenburgh C., Tanokura M., Denu J.M., Prolla T.A. (2010). Sirt3 mediates reduction of oxidative damage and prevention of age-related hearing loss under caloric restriction. Cell.

[28] Choudhary C., Kumar C., Gnad F., Nielsen M.L., Rehman M., Walther T.C., Olsen J.V., Mann M. (2009). Lysine acetylation targets protein complexes and co-regulates major cellular functions. Science.

[29] Kumar S., Lombard D.B. (2015). Mitochondrial sirtuins and their relationships with metabolic disease and cancer. Antioxid. Redox Signal..

[30] Kim H.S., Patel K., Muldoon-Jacobs K., Bisht K.S., Aykin-Burns N., Pennington J.D., van der Meer R., Nguyen P., Savage J., Owens K.M. (2010). SIRT3 is a mitochondrial localized tumor suppressor required for maintenance of mitochondrial integrity and metabolism during stress. Cancer Cell.

[31] Thomson D.M., Herway S.T., Fillmore N., Kim H., Brown J.D., Winder W.W. (2008). AMP-activated protein kinase phosphorylates transcription factors of the CREB family. J. Appl. Physiol..

[32] Jäger S., Handschin C., St-Pierre J., Spiegelman B.M. (2007). AMP-activatedproteinkinase (AMPK) action in skeletalmuscle via direct phosphorylationof PGC-1alpha. Proc. Natl. Acad. Sci. USA.

[33] Pillai V.B., Sundaresan N.R., Kim G., Gupta M., Rajamohan S.B., Pillai J.B., Samant S., Ravindra P.V., Isbatan A., Gupta M.P. (2010). Exogenous NAD blocks cardiac hypertrophic response via activation of the SIRT3-LKB1-AMP-activated kinase pathway. J. Biol. Chem..

[34] Hinchy E.C., Gruszczyk A.V., Willows R., Navaratnam N., Hall A.R., Bates G., Bright T.P., Krieg T., Carling D., Murphy M.P. (2018). Mitochondria-derived ROS activate AMP-activated protein kinase (AMPK) indirectly. J. Biol. Chem..

